# Hemoglobin Sickle-Beta-Thalassemia With an Acute Crisis

**DOI:** 10.7759/cureus.86078

**Published:** 2025-06-15

**Authors:** Hayato Tokuno, Kosuke Ishizuka, Takami Seki, Shintaro Kosaka

**Affiliations:** 1 Department of Hospital Medicine, Hiroo Hospital, Tokyo, JPN; 2 General Medicine, St. Marianna University, Kawasaki, JPN

**Keywords:** acute painful crisis, acute vaso-occlusive pain, hbs/β-thalassemia, sickle cell anemia, sickle cell disease

## Abstract

A 45-year-old Algerian man presented to our hospital in Japan with sudden onset of severe lower back pain. Peripheral blood smear showed target cells, and sickle cells constituting 4% of all erythrocytes, which led to the diagnosis of hemoglobin sickle-beta-thalassemia (HbS/β-thalassemia) with an acute crisis. The patient was hospitalized and treated with fluid therapy, continuous fentanyl infusion for pain management. He was discharged from hospital on Day 12, once the pain and anemia improved. Sickle cell disease is a rare disease in Japan, but with globalization, its incidence may increase in the future. It is important to know about acute painful crisis.

## Introduction

Sickle cell disease (SCD) is an inherited disorder caused by abnormal hemoglobin due to a point mutation in the hemoglobin beta-globin gene. There are subtypes of SCD such as sickle cell anemia (SCA), hemoglobin SC disease (HbSC), and hemoglobin sickle-beta-thalassemia (HbS/β-thalassemia) [1]. Under hypoxic conditions, as well as infection, dehydration, hypoxemia, physical exertion, cold exposure, and psychological stress, erythrocytes become deformed into a sickle shape and excessive destruction of the deformed erythrocytes results in hemolytic anemia. Also, deformed red blood cells lead to occlusion of capillaries and cause an acute painful crisis. In this report, we present a case of acute painful crisis secondary to vaso-occlusion in an Algerian man with HbS/β-thalassemia.

## Case presentation

**Figure 1:**
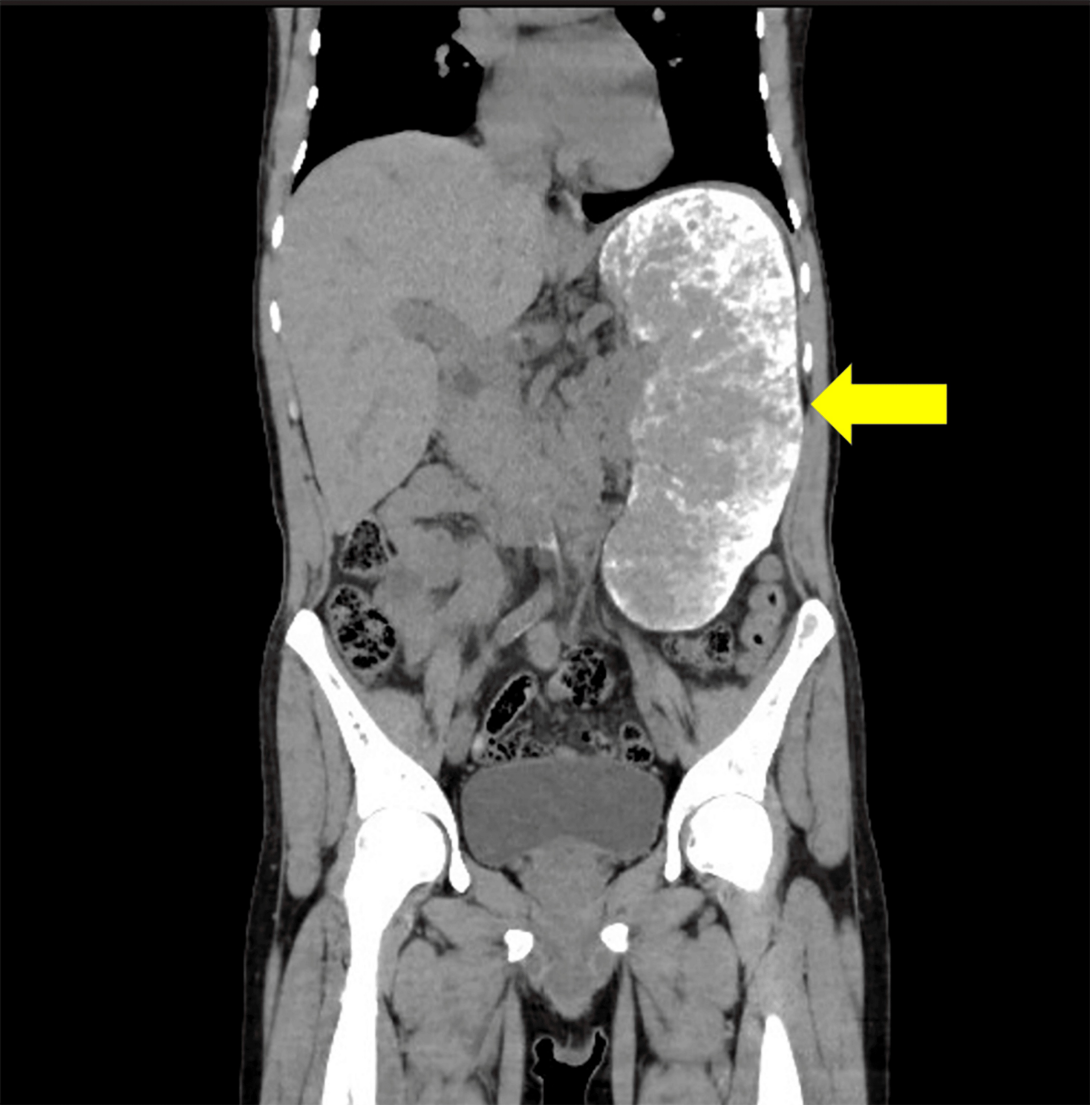
Computed tomography revealed marked splenomegaly, with diffuse high-density areas containing hemosiderin and calcification deposits.

**Figure 2:**
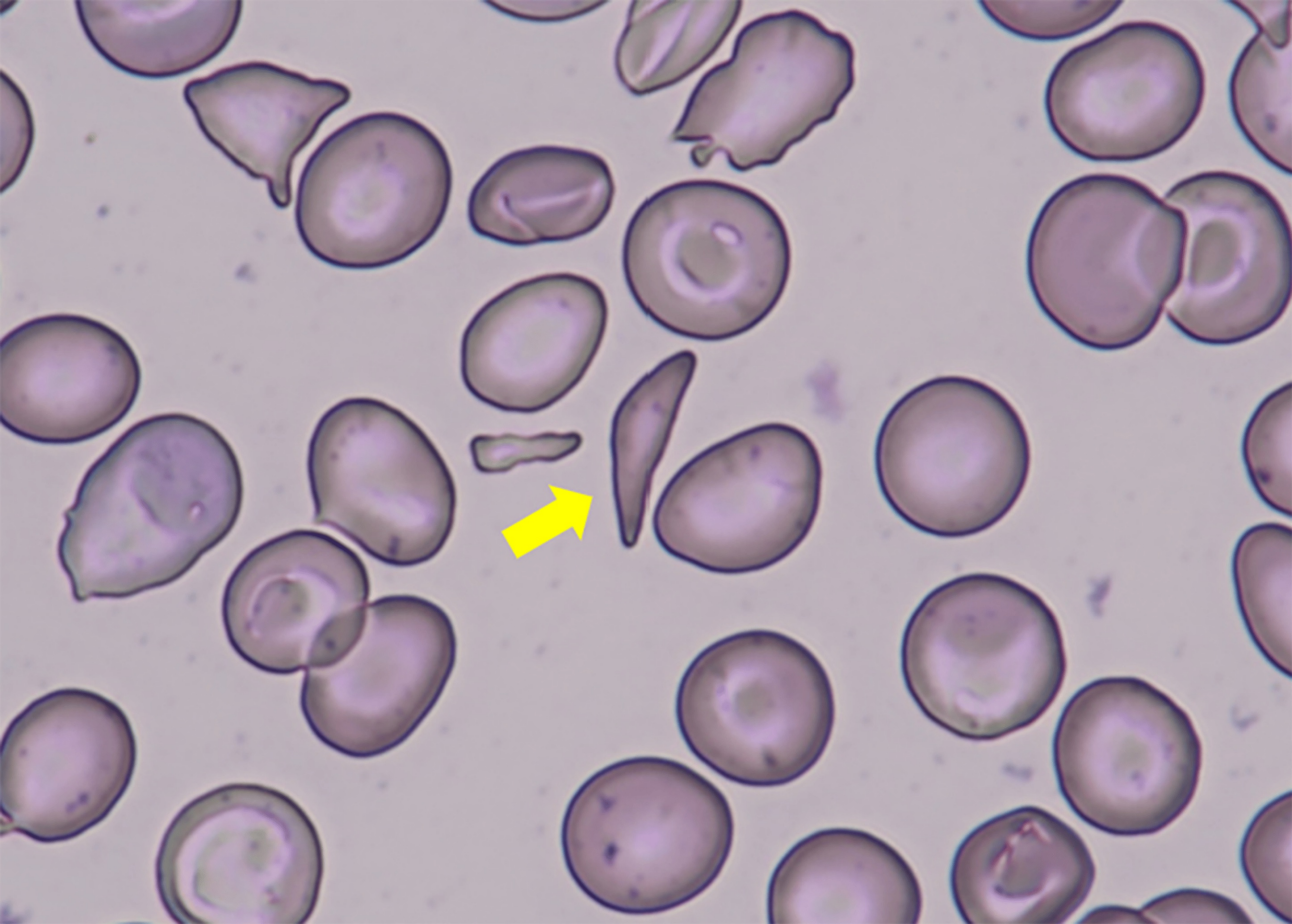
Peripheral blood smear (×1000 magnification) showed sickle cells constituting 4% of all erythrocytes.

A 45-year-old Algerian man with a history of sickle cell disease presented to our emergency department in Japan with sudden onset of severe lower back pain. He had a history of a common cold one week previously, and no trauma. He took no regular medications. He had been hospitalized at other medical facilities in the past due to similar pain. Physical examination revealed a temperature of 37.2°C, pulse rate of 109 beats/min, blood pressure of 141/86 mmHg, respiratory rate of 16 breaths/min, and oxygen saturation of 98% (room air). No muscle weakness or sensory disturbances were observed in the limbs. Laboratory tests showed an increased white blood cell count, decreased hemoglobin level and mean corpuscular volume, and elevated bilirubin with predominantly indirect bilirubin, lactate dehydrogenase and C-reactive protein levels; creatine kinase was within reference value (Table 1). ECG showed normal sinus rhythm at a rate of 89 bpm with no abnormalities. Computed tomography revealed marked splenomegaly (Figure 1). Peripheral blood smear showed target cells, and sickle cells constituting 4% of all erythrocytes (Figure 2), which led to the diagnosis of HbS/β-thalassemia with an acute crisis. The patient was hospitalized and treated with fluid therapy, continuous fentanyl infusion for pain management, and blood transfusion because of worsening hemolytic anemia. The continuous intravenous infusion of fentanyl was initiated at 2.5 µg/kg/h, gradually tapered by approximately 0.4 µg/kg/h every one to two days, and discontinued on Day 10. He was discharged from hospital on Day 12, once the pain and anemia improved.

**Table 1:** Laboratory results for the patient

Parameter	Patient Value	Reference value
White cell count	11.1 x 10^3 /μL	3.3 – 8.6 x 10^3 /μL
Hemoglobin	9.0 g/dL	13.7 – 16.8 g/dL
Mean corpuscular volume	66.0 fL	83.6 – 98.2 fL
Total bilirubin	5.8 mg/dL	0.4 – 1.5 mg/dL
Direct bilirubin	0.3 mg/dL	0.0 – 0.3 mg/dL
Creatine kinase	24 U/L	59 – 248 U/L
Lactate dehydrogenase	536 U/L	124 – 222 U/L
C-reactive protein	1.06 mg/dL	0.00 – 0.14 mg/dL

## Discussion

Sickle cell disease is a typical abnormal hemoglobinopathy. Sickle cell anemia, the most common form of sickle cell disease, refers specifically to the homozygosity of the βS allele. In populations of African ethnic origin, sickle cell anemia usually accounts for 70% of cases of sickle cell disease, and most of the rest are hemoglobin SC disease (HbSC disease) due to co-inheritance of the βS and βC alleles. The third and most common type of sickle cell disease is HbS/β-thalassemia, in which the βS allele is inherited along with the β-thalassemia allele [1]. The prevalence of sickle cell disease is high in large areas of sub-Saharan Africa, the Mediterranean basin, the Middle East and India. Although reliable estimates are scarce, neonatal estimates suggest that approximately 300,000 babies are born with sickle cell anemia each year, and the majority of these births occur in three countries: Nigeria, the Democratic Republic of the Congo, and India [2]. With globalization, it is expected that there will be more opportunities to see people from overseas in Japan. Symptoms complained of by people in sub-Saharan Africa, the Mediterranean, the Middle East, India, etc., especially in the case of pain, may be associated with acute vaso-occlusive pain in sickle cells. The main symptoms of sickle cell disease are vascular occlusion and hemolytic anemia. Acute vaso-occlusive pain is thought to be caused by red blood cells and white blood cells being trapped in the microcirculation, causing vascular occlusion and tissue ischemia. In a study of 555 sickle cell disease patients across the United States, Ghana, and Italy, the median age of the first pain crisis was four years old [3]. There is significant variability in the symptoms and clinical course of sickle cell disease. For example, in a collaborative study of sickle cell disease in the United States, 39% of 3,578 patients with sickle cell anemia had no pain episodes, while 1% had more than six episodes of pain per year [4]. In addition, 5.2% of patients with sickle cell disease experience three to 10 severe pains each year [5]. Infection, dehydration, hypoxia, exercise, cold stimuli, and stress are some of the triggers for pain attacks. In this case, there was a cold episode one week before the visit, which may have been associated with the trigger of the pain attack. These triggers should also be considered during medical history hearing. Acute pain is the most common reason for hospitalization in both adults and children, but it is more common in teens and young adults than in children. However, the majority of such events are managed at home with nonsteroidal anti-inflammatory drugs (NSAIDs) or non-prescription oral opioid analgesics without the involvement of the health care provider. Acute vaso-occlusive pain typically subsides spontaneously and does not lead to permanent organ damage, but from the patient's point of view, it is the most important complication. Additionally, in adult patients with sickle cell disease, the frequency of pain attacks was significantly associated with decreased survival [6]. In a typical episode of a pain crisis, patients complain of sudden pain in the lower back or one or more joints, or in one of the extremities [7]. Bone pain tends to be bilateral and symmetrical. This case is typical, with the patient presenting with acute back pain. We considered the differential diagnosis of acute back pain that could threaten life or function, but found no history of trauma or neurological findings. Contrast-enhanced CT showed no evidence of vascular dissection or occlusion. Therefore, we ruled out spinal cord compression due to fracture or tumor, and aortic dissection as causes of the back pain. The basic approach for acute painful crisis is to treat pain symptomatically by increasing the dose of non-opioid and opioid analgesics. Crises are often severe, requiring strong opioids such as morphine, fentanyl to alleviate symptoms. Careful monitoring is also important when using opioids [8]. In most patients, the pain crisis resolves within five to seven days. In patients with fever and respiratory symptoms, the confluence of acute thoracic syndrome should be considered. Acute chest syndrome is the most common cause of death in patients with SCD, and it is desirable to recognize it early and treat it appropriately [9]. In our case, the patient developed a fever after being admitted to the hospital, but no respiratory symptoms were observed. The pain improved spontaneously, so acute chest syndrome was not suspected.

## Conclusions

In areas where the prevalence of sickle cell disease is low, including Japan, there are few opportunities to encounter patients with sickle cell disease. However, with globalization, its incidence may increase in the future. It is important to raise sickle cell disease, recognizing variant forms like HbS/β-thalassemia, in the differential when seeing foreign patients. In the emergency setting, it is necessary to consider the differential diagnosis of complications such as acute painful crises and acute chest syndrome. Providing early and aggressive analgesia and being alert to signs of fever and respiratory symptoms are important for managing acute attacks.
